# Association of beverage consumption with subclinical atherosclerosis in a Spanish working population

**DOI:** 10.1038/s41598-023-33456-w

**Published:** 2023-04-20

**Authors:** Ainara Muñoz-Cabrejas, Martín Laclaustra, Pilar Guallar-Castillón, Raquel Sánchez-Recio, Estíbaliz Jarauta, José Antonio Casasnovas, Belén Moreno-Franco

**Affiliations:** 1 Instituto de Investigación Sanitaria Aragón, Hospital Universitario Miguel Servet, 50009 Zaragoza, Spain; 2 Department of Preventive Medicine and Public Health, Universidad de Zaragoza, C/Domingo Miral S/N, 50009 Zaragoza, Spain; 3 Department of Medicine, Psychiatry and Dermatology, Universidad de Zaragoza, C/Domingo Miral S/N, 50009 Zaragoza, Spain; 4 CIBERCV (CIBER de Enfermedades Cardiovasculares), 28029 Madrid, Spain; 5 Department of Preventive Medicine and Public Health, Universidad Autónoma de Madrid, 28029 Madrid, Spain; 6 CIBERESP (CIBER de Epidemiología y Salud Pública), 28029 Madrid, Spain; 7 IMDEA-Food Institute. CEI UAM+CSIC, 28049 Madrid, Spain

**Keywords:** Atherosclerosis, Nutrition

## Abstract

Beverages play a substantial role meeting water, calorie, and nutrient requirements; however, they are presented as being major contributors to the current obesity epidemic. Although, the relationship between beverage consumption and metabolic risk factors for cardiovascular disease (CVD) in adults has been frequently studied, its association with subclinical atherosclerosis is of increased interest. We studied the association of beverage consumption with the presence of peripheral subclinical atherosclerosis among Spanish workers. We performed a cross-sectional study of 2089 middle-aged males, with a mean age of 50.9 (SD 3.9), and without CVD, carried out in the Aragon Workers’ Health Study (AWHS). A food frequency questionnaire was used to measure beverage consumption of low-fat milk, coffee and tea (unsweetened), whole-fat milk, sugar-sweetened beverages, bottled fruit juice, artificially-sweetened beverages and 100% fruit juice. Atherosclerotic plaques were measured by ultrasound (in carotid arteries, and in femoral arteries). Atherosclerotic plaque was defined as a focal structure protruding ≥ 0.5 mm into the lumen, or reaching a thickness ≥ 50% of the surrounding intima-media thickness. As statistical analysis, we use logistic regression models, simultaneously adjusted for all beverage groups. As results, unsweetened coffee was the beverage most associated with peripheral subclinical atherosclerosis with an odds ratio (OR) of 1.25 (1.10–1.41), and 1.23 (1.09–1.40) 100g/day] for carotid, and femoral territories respectively. Moreover, subclinical atherosclerosis was positively associated with whole-fat milk [OR 1.10 (1.02–1.18) 100 g/day] in the femoral territory. The association was protective for low-fat milk in the carotid territory [OR 0.93 (0.88–0.99) 100g/day]. There was also a protective association with bottled fruit juices in the femoral territory [0.84 (0.74–0.94) 100g/day]. Our results suggest a detrimental association with the consumption of coffee, as well as with whole-fat milk and the presence of subclinical atherosclerosis. Therefore, an element of prudence excluding water and low-fat milk, must be applied when recommending beverage consumption.

## Introduction

Atherosclerosis is the pathological process that causes most cardiovascular disease (CVD). Lifestyle related factors, such as smoking, lack of physical activity, or an unhealthy diet are involved in its etiology^1–4^.

As part of the diet, beverages play a substantial role in meeting water requirements, and are an important source of calories as well as nutrients^4,5^. However, they are suspected of being one of the major contributors to the current obesity epidemic^6^. Thus, caloric free beverages -especially water- or those only containing beneficial nutrients, should be the primary beverages consumed^4,7^.

Current recommendations about the consumption of low-fat or fat-free dairy products rather than regular-fat^4,8^ have been questioned by different studies. Results regarding whole milk consumption are inversely associated with coronary arterial calcium progression especially in males^9^. They have been also associated with better cardiovascular profile such as lower systolic and diastolic blood pressure, and triglycerides (TG) levels, or higher high-density lipoprotein cholesterol (HDL-c), compared with non-consumption^10^.

Coffee might be related to delaying the atherosclerotic process in carotid arteries^11^. Furthermore, some studies have found a reduced risk of stroke with regular coffee intake^12^, while others have found an increased coronary heart disease (CHD) risk with a consumption of over 2 cups/day of espresso coffee^13^, but its consumption was not associated with a worse profile for plasma lipid concentrations.

The role of fruit juices is also the object of debate, even in the International Dietary Guidelines^4,7^, due to its reduced nutritional and fibre content and its higher caloric density, compared to whole fresh fruit. Instead, recent studies have found positive associations with a moderate intake of 100% fruit juice and pure fruit juice with lower risk of CVD, CHD, and stroke^14,15^.

Sugar sweetened beverages (SSBs) contain added caloric sweeteners such as sucrose, high sucrose corn syrup, or fruit-juice concentrates, and they are the largest contributors to added sugar intake^16^. Consumption of SSBs have also been shown to affect cardiovascular health^17^ with an increased risk of stroke, and myocardial infarction^18^. Conversely, artificially sweetened beverages (ASBs) have been proposed as a potential replacement for SSBs, but there is still scarce evidence on the benefits of this exchange^6,19,20^.

Although the relationship between beverage consumption and metabolic risk factors for CVD in adults has been frequently studied, the interest of the association between beverages and subclinical atherosclerosis is growing rapidly. Thus, we aim to study the association of non-alcoholic beverage consumption with the presence of subclinical atherosclerosis in the carotid and the femoral arteries.

## Materials and methods

### Study design and population

This cross-sectional analysis was carried out in a subsample of the Aragon Workers’ Health Study (AWHS), whose design and methodology have been previously described^21^. Briefly, the AWHS is a prospective cohort based on the annual physical examinations of 5678 workers belonging to an automobile assembly plant from Spain, with the aim of determining the risk factors for metabolic abnormalities and subclinical atherosclerosis. Between 2011 and 2014, a subgroup of 2616 participants aged 39–59 and free from CVD at baseline, attended extensive clinical examinations including subclinical atherosclerosis imaging, as well as an interview with questionnaires on diet, and lifestyles. We excluded females due to the small number (n = 132), and those with missing data on subclinical atherosclerosis (n = 316), and CVD risk factors (n = 79). The final sample comprised 2089 males (see Supplemental Fig. 1). The study was approved by the Clinical Research Ethics Committee of Aragon (CEICA). All participants provided written informed consent.

### Data collection

#### Diet assessment

Habitual diet during the preceding year of the interview was assessed using a semi-quantitative food frequency questionnaire (FFQ) previously validated in Spain^22^. This questionnaire is the updated version of the original FFQ, which collects information of 136 food items instead of the 118 food items initially contained, considering nine frequencies from “never or almost never” to “more than six times a day”. The updated version incorporates highly consumed foods in Spain and was designed to adapt to the Spanish diet and its evolution. The reproducibility and relative validity of this FFQ was assessed in the Predimed-Plus population, finding good reproducibility and a relative validity similar to those of FFQ used in other prospective studies^23^.

Consumption of milk (whole fat, semi-skimmed, and fat-free), decaffeinated coffee, caffeinated coffee and tea, 100% orange juice and other 100% fruit juices (with and without pulp, or from concentrate), bottled fruit juices (with and without added sugars), diet soda, and other artificially sweetened drinks, soft drinks, and milkshakes, were expressed in grams per day. Red, rosé, and white wine, beer and distillated drinks were expressed into daily grams of ethanol.

The semi-skimmed and the fat-free milks were combined into the category of “*low-fat milk*”. Unsweetened coffee, decaffeinated coffee, and tea were grouped to create a *“coffee and tea”* category. The “*whole fat milk*” category included just this item. A “*sugar-sweetened beverages*” category was created by summing up the other soft drinks, and milkshakes. Consumption of 100% orange juice and other 100% fruit juices were grouped to create a “*100% fruit juices*” category, while bottled fruit juices were grouped in a separate category as *“bottled fruit juices”*. In addition, the “*artificially sweetened beverages*” category included diet soda as well as artificially sweetened drinks.

#### Atherosclerosis imaging

The presence of plaques in the carotid and femoral arteries was assessed using a Philips IU22 ultrasound system (Philips Healthcare, Bothell, WA). Ultrasound images were acquired with linear high-frequency 2-dimensional probes (Philips Transducer L9-3, Philips Healthcare), using the Bioimage Study protocol for the carotid arteries^24^, as well as a specifically designed protocol for the femoral arteries^25^. Inspection sweeps were obtained on the right and left side of the carotid (common, internal, external, and bulb) as well as on the femoral territories. Plaque was defined as a focal structure protruding ≥ 0.5 mm into the lumen, or reaching a thickness ≥ 50% of the surrounding intima-media thickness. All measurements were analyzed using electrocardiogram gated frames corresponding to the end-diastole (R-wave)^26^.

#### Sociodemographic, clinical, and biological data

The main factory occupation is designed to allow for continuous car manufacturing, so workers are distributed in fixed shifts or rotating shifts. In night and rotating shifts (90% of the sample) most of the workers had elementary school education and belonged almost entirely to the manual labor workforce.

Age, sex, clinical, and laboratory data were obtained in the annual medical examination performed in the factory, including clinical data, BMI, blood pressure, medical history, and the current use of medication. Laboratory measurements were performed on blood samples collected in fasting conditions (> 8 h). Fasting serum glucose, TG, total cholesterol, and high-density lipoprotein cholesterol (HDL-c) were measured by spectrophotometry (Chemical Analyzer ILAB 650, Instrumentation Laboratory). Low-density lipoprotein cholesterol (LDL-c) levels were calculated using the Friedewald equation when TG were lower than 400 mg/dl. Arterial blood pressure was measured after a 5 min rest period with an OMRON M10-IT digital blood pressure monitor (OMRON Healthcare Co. Ltd., Japan), and we recorded the average of 3 consecutive automatic readings. Smoking habit was categorized as current smoking if the participant reported having smoked in the last year, former smoking if the participant had smoked at least 50 cigarettes in his lifetime, but not in the last year, and never smoking. Ever smoking (current and former) versus never smoking was used in the main analysis.

Arterial hypertension was defined as having systolic blood pressure ≥ 140 mmHg, or diastolic blood pressure ≥ 90 mmHg, or self-reported use of antihypertensive medication^27^. Dyslipidemia was defined as having total cholesterol ≥ 240 mg/dl, or LDL-c ≥ 160 mg/dl, or HDL-c < 40 mg/dl, or self-reported use of lipid-lowering drugs^28^. Diabetes was defined as fasting plasma ≥ 126 mg/dl or self-reported treatment with hypoglycemic medication^27^.

We assessed physical activity using the validated Spanish version^29^ of the questionnaire on the frequency of engaging in physical activity used in the Nurses’ Health Study^30^ and in the Health Professionals’ Follow-up Study^31^. A metabolic cost was assigned to each activity using the Ainsworth’s compendium for physical activities^32^. We computed the volume of activity performed by each participant, and it was multiplied by the time the participant reported practicing it. Summing up all activities, we obtained a value of overall weekly METs-h.

### Statistical methods

For descriptive porpoises, the mean of each beverage group was calculated including for those participants without consumption. The percentage of participants who did not consume was also reported.

The association between beverages and the presence of atherosclerotic plaques in carotid arteries (right and/or left, accounted jointly as one circulatory affected territory), and femoral arteries (right and/or left, accounted jointly as one circulatory affected territory) was examined using logistic regression. Separate analyses for each beverage group as well as mutually adjusted analyses taking into account all groups were performed. Models were adjusted for age, BMI, smoking status (ever smoker or never smoker), alcohol consumption (gr/day), hypertension, dyslipidemia, diabetes, and total METs-h/week.

The association of beverage groups with the presence of atherosclerosis was estimated using Odds Ratios (OR) and calculated in two ways: as 100 g per day of the beverage, which is a normative amount habitually used in nutritional epidemiology that allows for comparation with other studies; and per Standard deviation (SD) of consumption, reported as a log OR, in order to understand what beverage had a stronger association with the presence of atherosclerosis when considering its actual variation in the sample.

To better assess the relative importance of each beverage group on health, we translated ORs to years of arterial aging by dividing the coefficient of each beverage group by the coefficient of age, obtained in the same regression model.

The confounding variables were previously selected from those that in past research on diet and subclinical atherosclerosis had influenced the results, and those variables of dietary interest that historically may be potential confounders.

*P* values below 0.05 were considered statistically significant. R statistical software (v. 4.1.3) was used for the analysis.

### Ethical approval

The study was conducted according to the guidelines of the Declaration of Helsinki and approved by the central Institutional Review Board of Aragón (CEICA) (PI07/09).

## Results

### Descriptive analysis, beverage consumption and the percentage of participants without consumption

The 2089 participants had a mean age of 50.9 (SD: 3.9), and a mean BMI of 27.6 (SD: 3.3) kg/m^2^. Participants were ever-smokers in 77.1%, 37.4% had hypertension, 49.2% dyslipidemia, and 5.6% diabetes.

The beverage groups most frequently consumed were low-fat milk, coffee and tea (95% of consumption in this group was coffee), whole milk, and SSBs. There was a substantial number of participants who did not consume a specific beverage group, except for coffee. Within the rest of beverage groups, there was no consumption by at least one third of the participants in the sample (Table 1).

**Table 1:** Beverage consumption and the percentage of participants without consumption, among middle-aged men in the AWHS (N = 2089).

Component	Beverage consumption	
	Mean (SD) of gr/day	Percentage without consumption [count]
1. Low-fat milk (semi-skimmed)	155.6 (185.3)	39.5 [825]
2. Coffee and tea (unsweetened decaffeinated coffee, caffeinated coffee, and tea)	127.3 (76.7)	3.2 [67]
3. Whole-fat milk	68.5 (133.3)	66.4 [1387]
4. Sugar-sweetened beverages (soft drinks or milkshakes)	67.3 (128.3)	43.5 [909]
5. Bottled fruit juices	40.7 (82.5)	59.4 [1240]
6. Artificially sweetened beverages (diet soda or artificially sweetened drinks)	20.2 (84.4)	87.0 [1817]
7. 100% fruit juice (100% orange juice or other 100% fruit juice)	14.1 (43.6)	77.1 [1611]

Values are mean (standard deviation) or % [number]. Mean was computed including those without consumption.

### Association between 100 g/d of beverages consumed and the presence of plaques in peripheral arteries

The beverage most strongly associated with peripheral subclinical atherosclerosis was coffee [OR, 1.25 (1.10, 1.41), and 1.23 (1.09–1.40) per 100 g/day] for carotid, and femoral territories respectively. Moreover, subclinical atherosclerosis was associated with whole-fat milk consumption [OR 1.10 (1.02–1.18) per 100 g per day] in the femoral territory. On the contrary, low-fat milk showed a protective association [OR 0.93 (0.88–0.99) per 100 g/day] in the carotid territory. Bottled fruit juices also showed a protective association [0.84 (0.74–0.94) per 100 g/day] in the femoral territory (Table 2).

**Table 2:** Association between 100 g/d of beverages consumed and the presence of plaques in peripheral arteries among participants in the AWHS.

Association per 100gr				
Participants (N = 2089)	Carotid plaques OR (95%CI)		Femoral plaques OR (95%CI)	
	Separated analysis	Mutually adjusted analysis	Separated analysis	Mutually adjusted analysis
Low-fat milk (semi-skimmed)	0.94 (0.89, 0.99)*	0.93 (0.88, 0.99)*	1.01 (0.96, 1.07)	1.03 (0.98, 1.09)
Coffee and tea	1.23 (1.09, 1.39)***	1.25 (1.10, 1.41) ***	1.25 (1.10, 1.41)***	1.23 (1.09, 1.40)**
Whole-fat milk	1.05 (0.98, 1.12)	1.00 (0.93, 1.08)	1.10 (1.02, 1.18)*	1.10 (1.02, 1.18)*
Sugar-sweetened beverages	1.08 (1.00, 1.16)*	1.07 (1.00, 1.15)	1.08 (1.01, 1.17)*	1.08 (1.00, 1.17)
Bottled fruit juices	1.01 (0.90, 1.13)	1.01 (0.90, 1.13)	0.84 (0.75, 0.94)**	0.84 (0.74, 0.94)**
Artificially sweetened beverages	1.02 (0.91, 1.13)	1.03 (0.92, 1.15)	0.99 (0.89, 1.11)	1.00 (0.90, 1.12)
100% Fruit juice	1.01 (0.82, 1.25)	1.05 (0.84, 1.29)	0.94 (0.76, 1.17)	0.95 (0.76, 1.17)

AWHS, Aragon Workers’ Health Study; OR, odds ratio; CI, confidence interval.

N, total number of participants.

Asterisks denote *P* value: ***p ≤ 0.001, **p ≤ 0.01, *p ≤ 0.05.

^†^Adjusted for age, BMI, smoking status (ever smoker or never smoker), alcohol consumption (gr/day), hypertension, dyslipidemia, diabetes and total METs-h/week.

The mutually adjusted analysis included all beverage groups simultaneously in the same regression model.

### Association per standard deviation of beverages consumed and the presence of plaques in peripheral arteries

When studying the change per one SD, coffee was again the most influential group for peripheral subclinical atherosclerosis, with an absolute log OR per SD of 0.17 (0.08, 0.26), and 0.16 (0.06, 0.26) for the carotid, and the femoral territories respectively. The corresponding estimates for whole-fat milk obtained an absolute log OR of 0.12 (0.02, 0.22) for the femoral territory (Supplemental Table 1). Additionally, low-fat milk was − 0.13 (− 0.23, − 0.03) for the carotid territory and bottled fruit juices were − 0.14 (− 0.24, − 0.05) for the femoral territory.

### Association between 100 g/d and per standard deviation of beverages consumed and arterial aging

In the model with all the beverage groups mutually adjusted, the consumption of 100 g per day of coffee represented more than 2 years of arterial aging, both, for the carotid and femoral territories. The consumption of 100 g/day of whole milk aged femoral arteries about 1 year, while femoral arteries looked 1.9 years younger per 100 g/day of bottled fruit juice consumed. Additionally, the consumption of 100 g/day of low-fat milk prevented aging of carotid arteries about 0.7 years (Table 3). Considering the global influence in the sample, one SD of coffee consumption aged the arteries by 1.7 years, while bottled fruit juice prevented 1.5 years of aging in the femoral arteries, and low-fat milk prevented 1.3 years of aging in carotid arteries (Supplemental Table 2).

**Table 3:** Association between 100 g/d of beverages consumed and arterial aging among participants in the AWHS.

Association per 100gr—Arterial aging		
Participants (N = 2089)	Carotid plaques Log OR (95%CI)	Femoral plaques Log OR (95%CI)
Low-fat milk (semi-skimmed)	− 0.7 years or -9 months *	0.3 years or 4 months
Coffee and tea	2.3 years or 27 months ***	2.1 years or 26 months **
Whole-fat milk	0.0 years or 1 months	0.9 years or 11 months *
Sugar-sweetened beverages	0.7 years or 8 months	0.8 years or 9 months
Bottle fruit juices	0.1 years or 1 months	− 1.9 years or − 22 months **
Artificially sweetened beverages	0.3 years or 3 months	0.0 years or 0 months
100% Fruit juice	0.5 years or 6 months	− 0.6 years or − 7 months

AWHS, Aragon Workers’ Health Study; OR, odds ratio; CI, confidence interval.

N, total number of participants.

Asterisks denote *P* value: ***p ≤ 0.001, **p ≤ 0.01, *p ≤ 0.05.

^†^Adjusted for age, BMI, smoking status (ever smoker or never smoker), alcohol consumption (gr/day), hypertension, dyslipidemia, diabetes and total METs-h/week.

## Discussion

In this large epidemiological study, we found a consistent association between the consumption of coffee and the presence of subclinical atherosclerosis for the carotid as well as the femoral territories. Furthermore, we found a pernicious association for whole-fat milk in the femoral territory. In addition, a protective association was found for low-fat milk in the carotid territory, and for bottled fruit juice in the femoral territory. Thus, our results suggest that avoiding drinking coffee and consuming low-fat milk are options, which are not linked to negative outcomes and could potentially reduce the risk of subclinical atherosclerosis among middle-age men who were initially free of CVD.

Our results about low-fat milk and whole milk are in accordance with current international recommendations^4,8^, suggesting the benefit of consuming low-fat instead of whole milk. Nevertheless, current studies have reported inconsistent results about the harmful association of whole milk consumption and the development of CVD. On one hand, the study by Hidaka et al. has observed that low- and free-fat milk were associated with a reduction in atherogenic lipoprotein profile evidenced by lower plasma triglycerides and phospholipid levels, as well as being beneficial for TG/HDL-c and TG/LDL-c ratios^33^. On the other hand, as previously mentioned, the study of Sun et al. has observed that whole milk consumption improved cardiometabolic profile by lowering systolic and diastolic blood pressure, TG, as well as increasing HDL-c levels^10^.

Coffee is the most popular and widely consumed non-alcoholic beverages in the world^4^, and its role has been described to be beneficial on CVD incidence. Habitual coffee intake (more than three cups/day), has been shown to reduce the risk of heart failure on the Framingham Heart Study (FHS), the Cardiovascular Heart Study (CHS), and the Atherosclerosis Risk in Communities (ARIC) study^34^. Stevens et al. used machine learning based on random forest analysis to identify potential risk factors associated with CHD, stroke, and heart failure in the FHS. These results were later validated in the CHS and ARIC. Compared with those with no coffee consumption, the risk of heart failure was reduced respectively by 31% (HR, 0.69 [95% CI, 0.55–0.87]; *p* < 0.001) and by 29% (HR, 0.71 [95% CI, 0.58–0.89]; *p* < 0.001) in participants drinking 2 or 3 cups/day^34^. Moreover, Miranda et al.^35^, have reported that coffee intake decrease the odds for subclinical atherosclerosis measured by coronary artery calcium, among never smokers^35^. After stratifying by smoking status, the analysis revealed a lower OR for coronary calcification (CAC > 100) in never smokers who drank more than 3 cups/day [OR: 0.37 (95% CI, 0.15–0.91)], whereas among current and former smokers, the intake of coffee was not significantly associated with coronary artery calcium. Finally, a meta-analysis by Kim et al.^36^ has found that long-term coffee intake was inversely and significantly associated with CVD related mortality, with the lowest relative risk (RR) at intakes of 2.5 cups/day^36^.

However, not all previous studies report in the same direction. In accordance with our results, Grioni et al. found that consumption over 2 cups/day of Italian-style coffee was associated with increased risk of CHD [HR:1.37 (95% CI 1.03–1.82) for > 2–4 cups/day and 1.52 (95% CI 1.11–2.07) for over 4 cups/day (*p* trend < 0.001) compared to reference (< 1 cup/day)]. Coffee intake was not associated with plasma lipid changes^13^ in their study. It is important to highlight that differences in coffee elaboration and composition across southern Europe and other parts of the world may be responsible for the discrepancies. Indeed, consumption of unfiltered boiled coffee was associated with unfavourable changes in the lipid profile, and may cause a slight but significant rise in systolic blood pressure^37–39^. These unfavourable associations were mitigated with filtered coffee consumption, which is associated with a reduction of cardiovascular and general mortality, when compared to unfiltered coffee^40,41^. Apart from that, we cannot rule out the selection of the sample when studying older participants, instead of, in our case, where participants were middle-age men and free for CVD. With the available information, it still must be said that coffee consumption remains a question of debate within the scientific community.

So far, there is some controversy on the effect of fruit juice consumption. Our results suggest a reduction of subclinical atherosclerosis with bottled fruit juice consumption. In accordance with this, the study by Scheffers et al. showed that, compared with no consumption, up to 7 glasses/week of pure fruit juice consumption (defined as 100% fruit juice that can be both, fresh juice and bottled juice from concentrate) was significantly associated with reduced risk of CVD and CHD; and consumption of 1–4 and 4–8 glasses/week was significantly associated with a lower risk of stroke^15^. Furthermore, a recent meta-analysis by D’Elia et al. observed a non-linear inverse dose–response relationship between 100% fruit juice consumption and the risk of stroke (up to 200 ml/day), compared with no consumption, probably mediated by the decrease in blood pressure^14^. On the contrary, a recent meta-analysis of Pan et al.^42^ reported a significant association of 100% fruit juice intake with CVD mortality among highest category versus lowest category of 100% fruit juice consumption^42^. However, in our study we could not identify the effect produced by bottled fruit juice with or without added sugars. Thus, it may be necessary to know the composition of bottled fruit juice, such as type of fruit, added or artificially sugars, antioxidants, elaboration process, etc., in order to correctly evaluate these associations in the future.

In line with our results, the consumption of SSBs and ASBs have already been demonstrated to have harmful effects on cardiovascular health^43^. Consumption of SSBs was associated with increased risk of CVD, CHD, or stroke^18,43,44^. For example, results of a meta-analysis by Narain et al.^18^ suggest a greater risk of stroke (RR 1.13, 95% CI 1.02–1.24) and myocardial infarction (RR 1.22, 95% CI 1.14–1.30) with a one-serving per day increase in SSB consumption. When they evaluate high vs. low SSB consumption, the results suggest that there was a greater risk of myocardial infarction (RR 1.19, 95% CI 1.09–1.31). Moreover, their results suggest only a greater risk of stroke (RR 1.08, 95% CI 1.03–1.14) with a one serving per day increase in ASB consumption. When evaluating high vs. low consumption of ASB, there was a significantly greater risk of stroke (RR 1.14, 95% CI 1.04–1.26) and vascular events (RR 1.44, 95% CI 1.02–2.03)^18^. These results are in accordance with the study by Koning et al.^44^ in which an association between SSB consumption and CHD was found^44^. Additionally, results from the meta-analysis by Yin et al.^43^ suggest that 1 serving/day increment in SSB and ASB consumption was associated with an 8% and 7% CVD incidence and mortality, respectively.

Traditionally, diet quality has been based on solid food. However, beverages could play an important role in the onset and development of CVD. One of the main strengths of our study is that all beverages habitually consumed were considered. In addition, the “Mutually Adjusted Analysis” shows the results after adjusting the model for the rest of the beverages, eliminating the confusion that some beverages may exert on others. Additionally, the analyses were adjusted for traditional cardiovascular risk factors. Finally, the use of standardized protocols and high-quality data collection methods to obtain information on subclinical atherosclerosis stand out. Nevertheless, it has also several limitations. First, the cross-sectional design does not allow to establish causality nor the temporality of the associations found, although in this case, dietary intake was not modified by the presence of the subclinical disease, which was unknown by the participant. Second, our sample of females is too small to be analyzed separately. In addition, our sample comprised working males, all of whom worked in the same car assembly plant, as such, the results may not be directly generalized to the general population. Third, although the dietary assessment was conducted using FFQ by trained interviewers, we cannot rule out the presence of some misclassification^45^. However, the scientific literature supports that the FFQ is a feasible tool to evaluate food habits in epidemiological studies^46,47^. Fourth, even though we adjusted for the major potential confounders, residual confounding is still possible. Finally, other modifiers of the association between beverages consumption and subclinical atherosclerosis have not been considered.

In summary, prudence must be used when interpreting our findings and when making recommendation for CVD primary prevention. Excluding water and low-fat milk, the consumption of the other beverages within our study still require further debate, specifically when considering coffee consumption. While this debate remains, coffee consumption among middle-age men should not be encouraged.

## Conclusion

Our results suggest that, among middle-age men free from CVD, there was a detrimental association between the consumption of coffee and the presence of subclinical atherosclerosis. Moreover, a protective association of low-fat milk on the presence of subclinical atherosclerosis in carotid territories was observed.

## Supplementary Information

Supplementary Information 1.

Supplementary Information 2.

## Data Availability

Data described in the manuscript, code book, and analytic code will be made available upon request pending on request from the corresponding author. The data are not public due to ethical reasons.

## Abbreviations

ARIC study: Atherosclerosis Risk in Communities study
ASBs: Artificially sweetened beverages
AWHS: Aragon Workers’ Health Study
BMI: Body Mass Index
CAC: Coronary artery calcium
CEICA: Clinical Research Ethics Committee of Aragon
CHD: Coronary Heart Disease
CHS: Coronary Heart Study
CVD: Cardiovascular Disease
FFQ: Food Frequency Questionnaire
FHS: Framingham Heart Study
HDL-c: High-Density Lipoprotein Cholesterol
LDL-c: Low-Density Lipoprotein Cholesterol
METs: Metabolic Equivalents
OR: Odds Ratio
RR: Relative Risk
SD: Standard Deviation
SSBs: Sugar sweetened beverages
TG: Triglycerides
