# Supplementary material for: Association of beverage consumption with subclinical atherosclerosis in a Spanish working population
Source: Sci Rep. 2023 Apr 20;13:6509. doi: 10.1038/s41598-023-33456-w (PMC10119384; doi:10.1038/s41598-023-33456-w)
Supplement: Supplementary file 1 — Supplementary Information 1. [file 41598_2023_33456_MOESM1_ESM.docx]

**Supplemental Figure 1** Participant selection flow-chart.
