# Supplementary material for: Association of beverage consumption with subclinical atherosclerosis in a Spanish working population
Source: Sci Rep. 2023 Apr 20;13:6509. doi: 10.1038/s41598-023-33456-w (PMC10119384; doi:10.1038/s41598-023-33456-w)
Supplement: Supplementary file 2 — Supplementary Information 2. [file 41598_2023_33456_MOESM2_ESM.docx]

**Supplemental Table 1** Association per standard deviation of beverages consumed and the presence of plaques in peripheral arteries among participants in the AWHS.

| **Effect per SD** | | | | |
| --- | --- | --- | --- | --- |
| **Participants** (N = 2,089) | **Carotid plaques**  Log OR (95%CI) | | **Femoral plaques**  Log OR (95%CI) | |
|  | **Separated Analysis** | **Mutually Adjusted Analysis** | **Separated Analysis** | **Mutually Adjusted Analysis** |
| **Low-fat milk (semi-skimmed)** | -0.11 (-0.21, -0.02) * | -0.13 (-0.23, -0.03) * | 0.02 (-0.07, 0.12) | 0.06 (-0.04, 0.16) |
| **Coffee and tea** | 0.16 (0.06, 0.25) *** | 0.17 (0.08, 0.26) *** | 0.17 (0.07, 0.26) *** | 0.16 (0.06, 0.26) ** |
| **Whole-fat milk** | 0.06 (-0.03, 0.15) | 0.01 (-0.09, 0.10) | 0.12 (0.03, 0.22) * | 0.12 (0.02, 0.22) * |
| **Sugar-sweetened beverages** | 0.10 (0.01, 0.19) * | 0.09 (0.01, 0.18) | 0.10 (0.01, 0.21) * | 0.10 (0.0002, 0.20) |
| **Bottle fruit juices** | 0.01 (-0.09, 0.10) | 0.01 (-0.09, 0.10) | -0.14 (-0.24, -0.05) ** | -0.14 (-0.24, -0.05) ** |
| **Artificially sweetened beverages** | 0.01 (-0.08, 0.10) | 0.02 (-0.07, 0.11) | -0.01 (-0.10, 0.09) | 0.002 (-0.09, 0.10) |
| **100% Fruit juice** | 0.01 (-0.09, 0.10) | 0.02 (-0.08, 0.11) | -0.03 (-0.12, 0.07) | -0.03 (-0.12, 0.07) |

AWHS, Aragon Workers’ Health Study; OR, odds ratio; CI, confidence interval.

N, total number of participants.

Asterisks denote *P* value: *** *p≤0.001*, ** *p≤0.01, * p≤0.05.*

^†^Adjusted for age, BMI, smoking status (ever smoker or never smoker), alcohol consumption (gr/day), hypertension, dyslipidemia, diabetes and total METs-h/week.

The mutually adjusted analysis included all beverage groups simultaneously in the regression model.

**Supplemental Table 2** Association per standard deviation of beverages consumed and arterial aging among participants in the AWHS.

| **Effect per SD – Arterial aging** | | |
| --- | --- | --- |
| **Participants** (N = 2,089) | **Carotid plaques**  Log OR (95%CI) | **Femoral plaques**  Log OR (95%CI) |
| **Low-fat milk (semi-skimmed)** | -1.3 years or -16 months * | 0.6 years or 7 months |
| **Coffee and tea** | 1.7 years or 21 months *** | 1.7 years or 20 months ** |
| **Whole-fat milk** | 0.1 years or 1 months | 1.2 years or 15 months * |
| **Sugar-sweetened beverages** | 0.9 years or 11 months | 1.0 years or 12 months |
| **Bottle fruit juices** | 0.1 years or 1 months | -1.5 years or -18 months ** |
| **Artificially sweetened beverages** | 0.2 years or 3 months | 0.0 years or 0 months |
| **100% Fruit juice** | 0.2 years or 2 months | -0.3 years or -3 months |

AWHS, Aragon Workers’ Health Study; OR, odds ratio; CI, confidence interval.

N, total number of participants.

Asterisks denote *P* value: *** *p≤0.001*, ** *p≤0.01, * p≤0.05.*

^†^Adjusted for age, BMI, smoking status (ever smoker or never smoker), alcohol consumption (gr/day), hypertension, dyslipidemia, diabetes and total METs-h/week.
